# Analysis of chick (*Gallus gallus*) middle ear columella formation

**DOI:** 10.1186/1471-213X-10-16

**Published:** 2010-02-16

**Authors:** Jamie L Wood, Ami J Hughes, Kathryn J Mercer, Susan C Chapman

**Affiliations:** 1 Clemson University, Biological Sciences, Long Hall, Clemson, SC, 29634, USA; 2 The South Carolina Governor's School for Science and Mathematics, 401 Railroad Avenue, Hartsville, SC, 29550, USA

## Abstract

**Background:**

The chick middle ear bone, the columella, provides an accessible model in which to study the tissue and molecular interactions necessary for induction and patterning of the columella, as well as associated multiple aspects of endochondral ossification. These include mesenchymal condensation, chondrogenesis, ossification of the medial footplate and shaft, and joint formation between the persistent cartilage of the extracolumella and ossified columella. Middle and external ear defects are responsible for approximately 10% of congenital hearing defects. Thus, understanding the morphogenesis and the molecular mechanisms of the formation of the middle ear is important to understanding normal and abnormal development of this essential component of the hearing apparatus.

**Results:**

The columella, which arises from proximal ectomesenchyme of the second pharyngeal arch, is induced and patterned in a dynamic multi-step process. From the footplate, which inserts into the inner ear oval window, the shaft spans the pneumatic middle ear cavity, and the extracolumella inserts into the tympanic membrane. Through marker gene and immunolabeling analysis, we have determined the onset of each stage in the columella's development, from condensation to ossification. Significantly, a single condensation with the putative shaft and extracolumella arms already distinguishable is observed shortly before initiation of five separate chondrogenic centers within these structures. Ossification begins later, with periosteum formation in the shaft and, unexpectedly, a separate periosteum in the footplate.

**Conclusions:**

The data presented in this study document the spatiotemporal events leading to morphogenesis of the columella and middle ear structures and provide the first gene expression data for this region. These data identify candidate genes and facilitate future functional studies and elucidation of the molecular mechanisms of columella formation.

## Background

The columella consists of a persistent cartilaginous extracolumella laterally (comprised of three processes: supracolumella, extracolumella, and infracolumella arms inserting into the tympanic membrane) and an ossified shaft and footplate (also called the columella), which inserts into the inner ear oval window [1]. During columella endochondral ossification, bone replaces cartilage (replacement cartilage), unlike extracolumella (persistent cartilage), which remains throughout life. Identifying the mechanism of columella induction and patterning requires detailed knowledge of the morphological timing and developmental gene expression patterns. This study investigates the temporal events--including mesenchymal condensation, chondrogenesis, ossification of the medial footplate and shaft, and joint formation--between persistent and replacement cartilage. Determining the mechanism of normal and abnormal development of the middle ear is essential to understanding middle and external ear defects present in a subset of newborns with congenital hearing loss (~10%). In humans, these defects include first and second arch defects that result in external and middle ear defects and include, but are not limited to, microtia, aural atresia or stenosis, and congenital ossicle deformity. Given the common embryological origin of the middle and external ear, increasing severity of microtia is an indicator of the likelihood of middle ear defects [2-7].

The middle ear relays airborne sound waves from the environment to the fluid-filled inner ear. Sound vibrations on the tympanic membrane are transmitted and amplified across the air-filled middle ear cavity by the columella to the cochlear duct oval window. The lever action and motion of the footplate against the oval window transduce air-pressure fluctuations at the tympanic membrane to the cochlear endolymph, which, in turn, deform inner ear mechanoreceptor hair cells. Electrical signals then pass to the brain via the vestibulocochlear nerve (VIII).

There is still debate as to the origin of the columella in the chicken [8]. Fate mapping revealed that the columella originated from the mesenchephalon and anterior rhombencephalon-derived neural crest [9] and, more recently, from the more restricted domain of rhombomere 3-5-derived neural crest [10]. However, the footplate was never entirely composed of grafted quail cells, suggesting the possibility of a mesoderm contribution to the footplate. Supporting this hypothesis, Noden reported both neural crest and mesoderm contributions to the formation of a composite bony shaft and footplate [[8] and references therein].

The columella is the developmental equivalent of the second arch-derived mammalian stapes. The quadrate-articular joint forms the primary jaw articulation of all non-mammalian gnathostomes, including avians. In mammals, the primary articulation skeletal elements, together with the hyomandibula (columella of the chick and reptiles), was incorporated into the middle ear to form the chain of three auditory ossicles: malleus, incus and stapes (equivalent to the articular, quadrate and hyomandibula, respectively) [11]. Thus, the dentary-squamosal joint forms the secondary jaw articulation in mammals. Although developmentally equivalent to the stapes, the columella is functionally equivalent to all three mammalian ossicles [12].

Histological staining of whole mount and sectioned tissue was used to determine the timing of columella morphogenesis. In situ hybridization of marker gene expression and immunostaining further refined this temporal analysis. Our results indicate that the columella arises from a single proximal condensation, separating from the more distal second arch-derived hyoid skeleton. Multiple chondrogenic centers are identified in the putative shaft and extracolumella arms that are observed following initial mesenchymal condensation and the medial footplate and shaft, but not the extracolumella, are later ossified. Information from this study is essential to understanding normal and abnormal middle ear development and associated molecular mechanisms.

## Results

### Time course of columella condensation

Fate mapping studies have demonstrated that rhombomere 3-5 neural crest cells (ectomesenchyme) migrate to the second pharyngeal arch, where proximal cells form the columella and the distal population forms elements of the tongue skeleton and the cornua of the hyoid [10]. Epithelial-mesenchymal interactions result in mesenchymal condensation, followed by chondrogenesis and, finally, ossification of the medial columella shaft and footplate. However, little morphological data is available that describes the timing and positioning of the condensing ectomesenchyme that gives rise to the chick columella.

**Figure 1:**
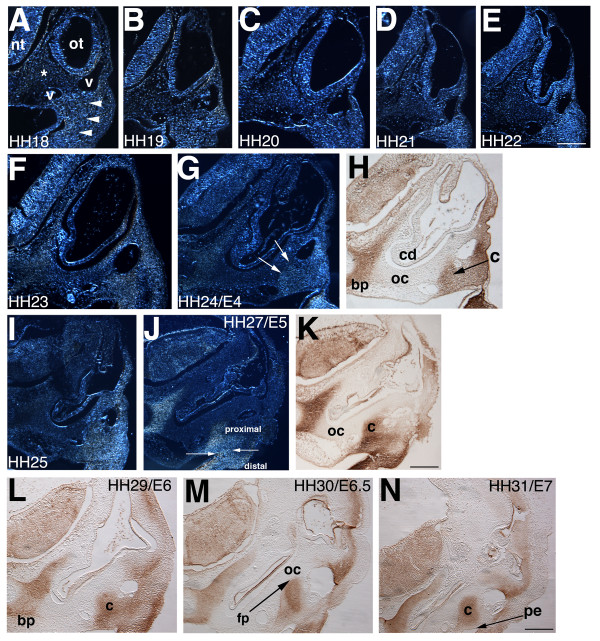
**The columella condensation arises from the proximal region of the second pharyngeal arch**. HH18-31 (E3-E7) embryos stained for peanut agglutinin lectin (PNA). Transverse paraffin sections at the level of the second arch, 10-12 μm. A-G, I, J darkfield; H, K-N brightfield. (A) PNA positive cells in second arch derived proximal and distal ectomesenchyme have a uniform appearance (arrowheads), ventral to blood vessels at HH18. Non-chondrogenic mesenchyme cells are PNA negative (asterisk). (B-E) HH19-22 sections show little change in the proximal region, with cochlear duct ventral extension. (F-H) Between HH23 and HH24, continued cochlear duct extension, with reshaping of the proximal condensation between the two blood vessels (arrows, G, H). (I-K) HH25-27, the columella condensation begins to separate from the more distal ectomesenchyme cells that gives rise to hyoid skeleton (opposing arrows, J). (L-M) The columella inserts into the otic capsule to form the oval window/footplate (arrowed). The pharyngeal endoderm (arrowed, N) will later form the tympanic membrane in apposition with surface ectoderm. The basal plate of the chondrocranium also exhibits PNA staining (G-N). Abbreviations: bp-basal plate of chondrocranium, v-blood vessels, cd-cochlear duct, c-columella, fp-footplate, nt-neural tube, oc-otic capsule, ot-otocyst, pe-pharyngeal endoderm. Scale bars: A-E 100 μm, F-L 150 μm, M, N 200 μm.

We histologically stained sectioned chick embryos from (Hamburger and Hamilton) HH14 to HH31 (E2-E7) with PNA (peanut agglutinin lectin), a marker of pre-chondrogenic progenitor cells in condensations [13-15]. PNA binds galactose-containing surface moieties--specifically cell surface glycoproteins or proteoglycans--and labels the columella and hyoid condensations of the second pharyngeal arch and basal plate condensation of the chondrocranium (Figure 1). Sections were analyzed using darkfield microscopy to enhance the contrast between PNA-labeled and non-labeled cells.

PNA labeling is undetectable between HH14 and HH17 (not shown). At HH18, labeled ectomesenchyme cells can be observed in the proximal and distal second arch (Figure 1A). A sharp line between the ventral surfaces of the two head vessels (anterior cardinal and dorsal aorta) denotes the boundary between labeled pre-chondrogenic (ectomesenchyme) and unlabeled non-chondrogenic cells (possibly head mesoderm) (Figure 1A-E). Between HH18 and HH22, the head grows rapidly and the cochlear duct undergoes ventral extension. From HH23 to HH24, the position of the PNA-labeled cells changes relative to the cranial blood vessels, with labeled cells detected more dorsally--between, rather than below, the vessels (arrowed, Figure 1F-H). PNA does not stain the otic capsule condensation, probably because of the presence of sialic acid residues that mask PNA labeling, but this conjecture remains to be verified (Figure 1H) [16,17]. Sialic acid residues interfere with PNA labeling in mouse Meckel's cartilage and in the nasal septum [18]. For our study, the labeled columella adjacent to the unlabeled otic capsule is beneficial in distinguishing between these two structures. Between HH25 and HH27 (E5), the columella condensation separates from the more distal ectomesenchyme (Figure 1I-K) of the tongue and hyoid skeleton [10]. Remodeling of the columella condensation continues from HH29 to HH31 (E6-E7) with overt chondrogenesis first detected with the use of Alcian blue staining at E7. The footplate of the columella directly contacts the otic capsule (Figure 1L-N). The extracolumella arms extend toward the pharyngeal endoderm/surface ectoderm interface of the still unformed tympanic membrane, into which the extracolumella arms will insert.

Of note is that the columella condensation separates from the more distal hyoid condensation at E5 (Figure 1J). The mechanism of the remodeling and separation of the proximal and distal condensations to produce a condensation of the correct size and shape is unknown, but it likely includes cell proliferation, cell migration, and cell shape changes.

### Chondrogenesis occurs earlier in replacement cartilage than in persistent cartilage of the columella

**Figure 2:**
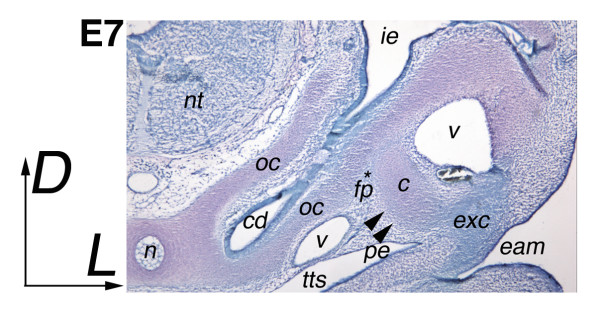
**Morphology of the ear-forming region**. E7 Toluidine Blue section, 12 μm showing the relationship of otic capsule to columella and extracolumella elements. The section is diagonal to include columella and extracolumella, with the latter more posteriorly situated relative to the notochord than the columella. Chondrogenic tissue stains pink, with the otic capsule, columella and cartilage around the notochord all stained. The extracolumella condensation appears blue, indicating that chondrogenesis has not yet begun. Note the perichondrium surrounding the columella (arrowed), and footplate (asterisk), which are also blue. Abbreviations: cd-cochlear duct, eam-external auditory meatus, fp-footplate, ie-inner ear, nt-neural tube, n-notochord, oc-otic capsule, pe-pharyngeal endoderm, tm-tympanic membrane, tts-tubotympanic sulcus, v-blood vessels.

The morphology of the ear-forming region is demonstrated at E7 with Toluidine Blue staining (Figure 2). The columella condensation is oriented ventrolaterally relative to the inner ear cochlear duct, with the footplate inserting into the otic capsule, and the extracolumella extending laterally toward the future tympanic membrane. Pharyngeal endoderm and surface ectoderm of the external auditory meatus will form the tympanic membrane, with extracolumella and infracolumella arms inserted. The otic capsule and columella are pink, an indication of chondrogenesis, but the extracolumella is still blue, indicating that the extracolumella is still a pre-chondrogenic condensation that lacks chondrogenic cells.

**Figure 3:**
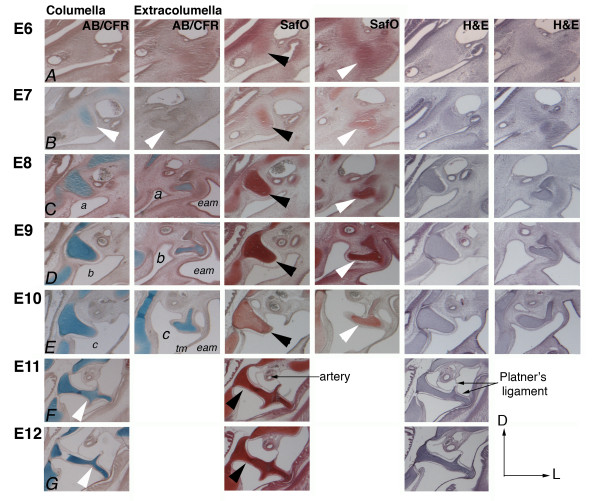
**Timeline of chondrogenesis of the columella condensation**. Transverse paraffin sections at the level of the second pharyngeal arch, 12 μm. Counterstaining of sectioned heads, E6-E12 with Alcian Blue/Chlorantine Fast Red (AB/CFR), SafraninO (SafO), and Hematoxylin and Eosin (H&E). Columella columns 1, 3, 5; extracolumella columns 2, 4, 6. The columella is marked by black arrowheads in SafO sections (column 3), the extracolumella by white arrowheads (column 4). (A) Initially, few chondrocytes are detected by Alcian Blue in the columella at E6 and none in the extracolumella. (B) By E7, the perichondrium is forming around the condensation (white arrowhead), with Alcian Blue stained chondrocytes in the columella, but absent in the extracolumella. (C) Note the expanding tubotympanic sulcus (a) and ingressing external auditory meatus, which appose to form the tympanic membrane at E8. (C-E) E8-E10, staining refines and the extent of chondrogenesis in the columella and extracolumella is evident. Note the dorsal expansion of the tubotympanic sulcus forming the pneumatic middle ear cavity around the columella (labeled a, b, c - columns 1/2) (F, G). E11 and E12 counterstaining detects chondrocytes of both persistent and replacement cartilage. The columella is now transversely oriented with respect to the notochord. The supracolumella arm is dorsal, and infracolumella arm ventral. Abbreviations: eam-external auditory meatus, tm-tympanic membrane.

To determine the timing of chondrogenesis, sectioned chick tissue from E6-12 was stained and examined using Alcian Blue/Chlorantine Fast Red (AB/CFR), Safrinin O (SafO) and Hematoxylin & Eosin (H&E) (Figure 3). SafO reveals the developmental progression of columella (black arrowheads) and extracolumella (white arrowheads) (columns 3/4). Alcian Blue labels chondrogenic cells (column1/2) from E7. The columella shaft is stained, whereas the extracolumella shows little sign of chondrogenesis and matches the Toluidine Blue staining (Figure 2). The perichondrium begins to envelop the condensation (row B). Alcian blue staining is in the extracolumella beginning from E8 and gradually becomes more defined (row C). The pharyngeal endoderm of the tubotympanic sulcus and ectoderm of the external auditory meatus gradually become apposed to form the tympanic membrane between E8 and E10 (rows C-E). The dorsal expansion of the tubotympanic sulcus endoderm, beginning from E8, creates the pneumatic middle ear cavity (Jaskoll and Maderson, 1979). The endoderm lines the middle ear cavity and columella, supporting the ossicle between the oval window and tympanic membrane (E8-E12). The cavities of opposing ears are directly connected, creating an interaural canal connected to the nasopharynx via a common midline canal that enables sound localization and equalization of air pressure between the middle ear cavity and the external auditory meatus (labeled a, b, c, columns 1/2) [19]. At E11 and E12, counterstaining detects chondrocytes of both replacement cartilage (columella), which is to be replaced by bone, and persistent cartilage (extracolumella), which will remain throughout life (rows F and G). The orientation of the columella has changed during development and is now transverse to the notochord; this change facilitates visualization of the whole columella in a single section, whereas at earlier stages the extracolumella was posteriorly angled. The three arms of the extracolumella--the supracolumella arm dorsally, the extracolumella arm pushing against the endoderm of the tympanic membrane, and the long, flexible infracolumella arm--insert ventrally into the tympanic membrane (rows F and G). Platner's ligament (also called the squamosal-columella ligament) has two identifiable tendons by E11 (row F and G).

To summarize, the replacement cartilage (columella footplate and shaft) undergoes chondrogenesis earlier than the extracolumella, which remains cartilaginous throughout life. The position of the columella becomes transverse with respect to the notochord by E11, whereas it is initially angled posteriorly relative to the notochord. Columella development is dynamic, and several days are required for it to span the middle ear cavity, with footplate insertion into the oval window and formation of the tympanic membrane.

### Multiple chondrogenic centers within the columella condensation revealed by whole mount staining

**Figure 4:**
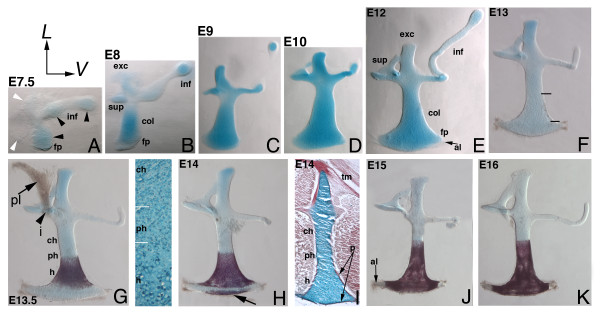
**Whole mount columella extracted from embryo**. Alcian Blue/Alizarin Red S staining of the columella (col), E7-E16. (A) The columella at E7.5 with chondrogenic centers in the footplate/shaft and two more in the infracolumella (inf) arm (black arrowheads). The supracolumella (sup) and extracolumella (exc) condensations (white arrowheads) have no chondrocytes. (B) By E8 five condensation centers can be identified: columella shaft, supracolumella, extracolumella, and two in the infracolumella. (C, D) During E9/10 continued chondrogenesis and growth occurs. (E) At E12, the extent of the delicately sculpted infracolumella arm is visible. (F) At E13, pre-hypertrophic chondrocytes appear in the shaft of the columella (area between two black lines). (G) E13.5, Alizarin Red S staining is detected in the periosteal bony collar of the shaft. Platner's ligament is inserted onto the supracolumella/extracolumella base (arrowhead). The adjacent section shows chondrocytes, pre-hypertrophic, and hypertrophic chondrocyte progression. (H) A second periosteum becomes apparent at E14 in the base of the footplate (arrowed). (I) A section with periosteum labeled red (arrowed). The extracolumella arm inserts into the tympanic membrane (tm). (J) By E15 the periosteum regions merge, with the annular ligament negative (arrowed). (K) E16, note Alizarin Red S staining compared to earlier stages in the shaft (compare H-K). Abbreviations: al-annular ligament, ch-chondrocytes, fp-footplate, h-hypertrophic chondrocytes, i-insertion point, p-perichondrium/periosteum, ph-pre-hypertrophic chondrocytes, pl-Platner's ligament.

To determine the gross morphological development of the middle ear, we stained whole chick embryo heads with Alcian Blue and Alizarin Red S from E7-16 (Figure 4). E7.5 is the earliest stage at which we were able to extract intact condensations from labeled heads. These condensations have a distinct structure, with five regions identifiable in the condensation as a whole (Figure 4A, arrowheads). However, only the shaft and two regions of the infracolumella label with Alcian Blue, indicating separate chondrogenic centers (black arrowheads). The supracolumella and extracolumella arms do not develop distinguishable chondrocytes until E8 (white arrowheads), when all three extracolumella arms have chondrogenic centers. The shaft elongates, with chondrocytes labeled throughout the condensation (Figure 4C-F). The footplate at the base of the shaft (Figure 4B), has unlabeled annular ligament precursor cells identifiable by E12 (Figure 4E). Only the extracolumella arm and long, flexible infracolumella arms insert into the tympanic membrane; the supracolumella arm does not insert into the tympanic membrane but, together with an attachment to Platner's ligament, instead stabilizes the columella with its spatula-like shape, acting as a brace, [20]. By E13 a change in the morphological appearance of the shaft cells is evident (Figure 4F), indicating that the next stage in development has begun.

### Two regions of periosteum development are evident in the columella

By E13.5 the periosteum surrounds the columella shaft, taking up Alizarin Red S stain (Figure 4H). Cells exhibit the enlarged pre-hypertrophic phenotype (mid-shaft) and hypertrophic chondrocytes (base and footplate), whereas only immature chondrocytes are detected in extracolumella persistent cartilage (Figure 4G, and section inset). One tendon of Platner's ligament was retained in this preparation (Figure 4G).

Unexpectedly, a separate periosteal layer appears in the base of the footplate at E14 (Figure 4H, I, arrows). The two periosteal regions become continuous by E15 (Figure 4J). Timing of osteoblast invasion into the matrix at E15 is in agreement with the timing proposed by Jaskoll and Maderson (1979), but formation of the periosteum at E13.5 occurs earlier than previous studies have suggested. The bony columella will later form a hollow interior, with the surface tissue providing the strength necessary for movement in the oval window (not shown) [19]. The fibrous annular ligament, which holds the columella in the oval window of the cochlear duct, surrounds the bony footplate and is, as expected, negative for staining (Figure 4G-K).

The periosteum extends up the shaft toward the extracolumella during development as the mid-shaft pre-hypertrophic cells mature, with organization reminiscent of a growth plate (Figure 4H-K). Once all chondrocytes are transformed, this mid-shaft region forms the joint region between the replacement and persistent cartilage. The bony columella has the rigidity required for the movement of the footplate within the oval window, while also allowing the flexible extracolumella arms that are inserted into the tympanic membrane to receive and amplify sound waves as the tympanic membrane distorts in response to sound vibrations. The extracolumella arms that are inserted into the tympanic membrane cover a large surface area, whereas the footplate has a smaller area (11:1 ratio) [19], resulting in amplification of sound via increased pressure at the oval window.

Thus, staining of extracted columellas demonstrates that the condensation has a distinct shape before the onset of chondrogenesis, which continues to grow and refine during development. Chondrogenesis occurs in multiple centers within the condensation and progresses spatiotemporally from the shaft toward the extracolumella region. The formation of two separate Alizarin Red S stained periosteum (bony collar) regions in the shaft and footplate was unexpected. Establishing timing of these events is an important precursor to understanding the molecular mechanisms responsible for bone formation.

### Marker gene expression analysis reveals the timing of chondrogenesis and chondrocyte differentiation

Marker genes were analyzed in the developing middle ear region from E5, before chondrogenesis could be identified, and continued from E7 to E10 to determine the changes in expression as chondrogenesis progressed. Only the otic capsule/columella region is shown; any differences in the extracolumella will be noted.

#### Sox9

**Figure 5:**
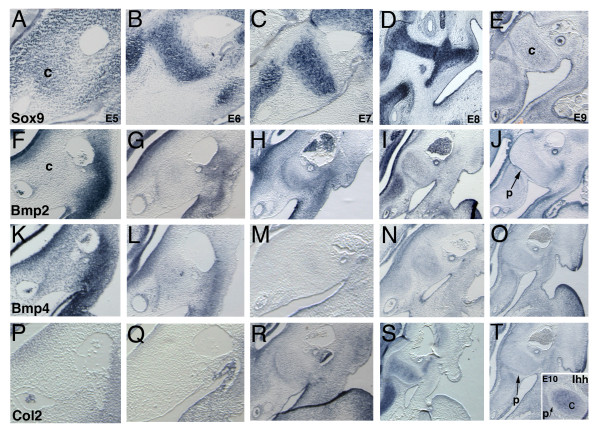
**ISH analysis during columella development**. Transverse sections of paraffin embedded embryos showing the columella from E5-E10, 10-12 μm. (A, B) Sox9 is expressed in the otic capsule and columella condensations. (C) Expression remains strong at E7, with the labeled extracolumella at E8 (D), but dramatically downregulated, with weak mosaic expression at E9 (E). (F, G) Bmp2 is not expressed in the columella at E5/6. (H, I) Bmp2 briefly upregulates during chondrogenesis, with transcripts also detected in the perichondrium (H-J) (J) Expression continues to weaken, except for the perichondrium (arrowed). (K-M) Bmp4 has weak, widespread expression at E5, downregulating at E6 and undetectable at E7. (N-O) Low expression levels recur at E8/9. (P, Q) Col2 is not expressed in the columella condensation and only weakly expressed in the otic capsule at E5/6. (R) Col2 upregulates in the otic capsule, columella, and extracolumella at E7, strengthening at E8 (S), rapidly clearing at E9 (T). Ihh up regulates at E10 in the otic capsule and columella, but not the perichondrium (inset, p, arrowed).

The columella is situated between the cochlear duct, two blood vessels of the head, and the pharyngeal endoderm. A known activator of Col2 [21], Sox9 is detected in chick neural crest cell precursors as early as HH10 [22]. Sox9 expression is sufficient and required for chondrocyte differentiation in both persistent and replacement cartilage [23]. At E5, strong Sox9 expression is all pre-chondrogenic cells--including the otic capsule, the columella, the extracolumella and distal ectomesenchyme of the second arch (Figure 5A). Expression becomes more restricted at E6 as cells within the condensations become more closely associated (Figure 5B). At E7, strong expression labels the condensations (Figure 5C), with the extracolumella arms taking shape and inserting into the forming tympanic membrane at E8 (Figure 5D). Expression is dramatically downregulated, although low levels are detected in a mosaic pattern at E9 in the otic capsule and the columella (Figure 5E). By E10 expression in the cartilage matrix is largely undetectable (not shown).

#### Bmp2

Application of recombinant BMP2 protein induces cartilage and, at high doses, bone [24], suggesting that it plays an important role in condensations. Bmp expression patterns have not been previously described for the chick middle ear. At E5 and E6, expression is minimal in the condensation, but it is higher in the nascent perichondrium at E6 and strong in the lateral and distal ectomesenchyme of the second arch (Figure 5F, G). With the onset of chondrogenesis in the columella at E7, Bmp2 transcript levels in the columella increase (Figure 5H), albeit transiently, with weak mosaic expression in the columella cartilaginous matrix at E8 (Figure 5I). However, expression persists in the perichondrium at E8 and E9 (arrowed, Figure 5J). Overall, perichondrium expression is stable beginning from E6, with a brief upregulation in the columella matrix at E7 as chondrogenesis occurs, and then downregulating again by E9.

#### Bmp4

Widespread Bmp4 expression occurs at E4 (not shown) and E5 (Figure 5K). However, by E6 expression weakens (Figure 5L) except for the lateral and distal cells of the second arch. Almost complete downregulation occurs in the columella by E7 as chondrogenesis begins (Figure 5M). This expression is the opposite of the observed Bmp2 expression. Expression increases slightly at E8 but remains weak at E9 (Figure 5N, O). The functional significance of opposing Bmp2 and Bmp4 gene expression at the onset of chondrogenesis is the subject of ongoing studies.

#### Col2 and Ihh

Col2 expression is undetected at E5 and E6 in the columella condensation (Figure 5P, Q), an indication that chondrogenesis has yet to occur. Cells in the columella core upregulate Col2 expression at E7, marking the onset of chondrogenesis (Figure 5R). The otic capsule and the extracolumella also have expression, which strengthens further by E8 (Figure 5S) while the perichondrium remains negative. Expression is dynamic and downregulates, just as for Sox9, by E9 (Figure 5C) with concomitant upregulation of Ihh expression (Figure 5 inset) at E10. Before E10, Ihh expression is not detected in the otic capsule, columella or extracolumella (not shown). Upregulation at E10 indicates the beginning of chondrocyte differentiation with the transition of chondrocytes toward hypertrophy.

We show that upregulation of Bmp2 and Col2 gene expression at E7 is an indication of chondrogenesis. Sox9 and Col2 expression downregulates at E9, with Ihh upregulation following at E10, indicating that chondrocyte differentiation has begun.

### Collagen1 labels periosteum formation in columella replacement cartilage

**Figure 6:**
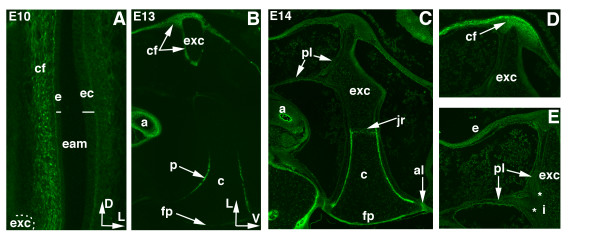
**Collagen1 is a marker of replacement cartilage and collagen muscle fibers**. Transverse 10-12 μm sections, COL1 immunostaining at E10, 13, and 14. (A) High magnification image of the tympanic membrane and external auditory meatus. Collagen fibers (cf) underlie the medial tympanic endoderm (e), forming part of Platner's ligament (pl). The more lateral ectodermal layer of the tympanic membrane is thicker than the simple squamous epithelium of the endoderm (bars). The insertion site of the extracolumella process is indicated by a dotted line (bottom left). (B-E) Lateral is to the top and ventral to the right. (B) At E13, COL1 in the bony collar of the shaft, the periosteum (p), but not the perichondrium of the footplate (fp). Collagen fibers underlie the tympanic endoderm, extending around the extracolumella (exc). Large numbers of collagen fibers are detected in the external ophthalmic arterial (a) wall. (C) At E14, COL1 labels the periosteum of both footplate and shaft. Note the joint region in the shaft (arrowed), and a band of fibers in the annular ligament (al). (C, D) Labeled collagen fibers underlying the endoderm at E14. (C, E) The two tendons of Platner's ligament insert onto the base of the supracolumella/extracolumella arms (pl, arrowed). Abbreviations: a-external ophthalmic artery, c-columella, eam-external auditory meatus, ec-ectoderm, i-insertion point, jr-joint region.

At E10, no COL1 is detected in the columella (Figure 6A); however, collagen fibers below the endoderm of the tympanic membrane are labeled (Figure 6A, D). The endodermal epithelium that lines the medial surface of the tympanic membrane is composed of a single layer of simple squamous epithelium. The ectodermal epithelium, situated laterally, is thicker and is composed of keratinizing squamous epithelium [1]. The endoderm of the tympanic membrane bulges outward into the external auditory meatus because the extracolumella arm pushes against the collagen fibers on the inner endodermal surface, producing the drum-like tension of the membrane (Figure 6B-D). Thus, the columella is under tension between the tympanic membrane and the oval window.

COL1 expression appears in the periosteum of the columella shaft at E13 (Figure 6B), forming a bony collar. Alizarin Red S staining is similarly detected in the bony collar at E13.5 (Figure 3H). A new domain of COL1 is detected in the footplate at E14 (Figure 6C), again matching Alizarin Red S staining (Figure 3I). A band of COL1 is detected across the joint region between replacement and persistent cartilage (Figure 6C). Persistent cartilage cells appear closely packed, whereas those under the collagen band are enlarged and have the typical appearance of hypertrophic cells, suggesting that this is the joint region between prehypertrophic and hypertrophic cells. We speculate that, as the periosteum extends towards the extracolumella, these prehypertrophic cells become hypertrophic, but this speculation remains to be substantiated. It will be interesting to determine if the band of collagen is fixed in place, or if position changes as the periosteum extends toward to the extracolumella.

The band of collagen fibers in the annular ligament (Figure 6C) extends around the ligament, stabilizing the footplate in the oval window and providing a cushioning effect during the motion of the columella. The squamosal-columella ligament is split into several tendons (not shown). The muscle portion, which contracts, lies outside the tympanic membrane and attaches to the squamosal bone. Several tendons cross the middle ear cavity, attaching to the edges of the tympanic membrane (not shown) [19]. Two tendons that form Platner's ligament are also apparent (Figure 6C). They insert onto the extracolumella; one attaches posteriorly at the base of the extracolumella between the supra- and infracolumella processes and extends toward the external ophthalmic artery. The other tendon is attached at the base of the extracolumella and extends toward the collagen fibers that underlie the endoderm of the tympanic membrane (Figure 6B, D, and 6C, E).

Our results demonstrate that two separate regions of the periosteum arise, one a bony collar surrounding the shaft, and a second at the base of the footplate. Later, the periosteum regions become continuous. COL1 acts as a marker of periosteum formation and the onset of ossification of the footplate and delineates the boundary region between prehypertrophic and hypertrophic cells in the columella shaft.

## Discussion

### The onset of condensation formation occurs soon after neural crest migration

Early skeletogenesis is roughly divided into four phases: migration, epithelial-mesenchymal interaction, condensation, and chondrocyte differentiation/maturation. Migration of rhombomere-derived neural crest cells to the proximal second pharyngeal arch occurs between stages 11 and 14. The nascent columella condensation is first detected by PNA labeling at stage 18 (E3), after which elongation of the cochlear duct and growth of the surrounding tissue repositions the nascent columella condensation, separating it from the more distally situated second arch ectomesenchyme. The distal hyoid skeleton consists of the basihyal (part of the basibranchial) and ceratohyal (entoglossal and hyoid cornua) and forms part of the tongue skeleton [25]. Thus--like the first arch condensation mechanism, in which a single element separates, giving rise to the multiple lower jaw elements--a single second arch mesenchymal condensation gives rise to several distinct skeletal elements[26].

The proximal pharyngeal endoderm of the second arch directly contacts the nascent columella, suggesting an inducer/responder relationship between these tissues (an epithelial-mesenchymal interaction) in the induction of the columella condensation. A role for pharyngeal endoderm in patterning craniofacial structures is demonstrated by the sequential anterior-to-posterior endoderm ablation domains that results in the loss of associated skeletal elements [27]; endoderm removal in the vicinity of the second arch results in loss of cerato- and epibranchial elements, whereas addition of homotopic grafts result in duplications. The effect of these ablations on the columella were not analyzed (Ruhin, personal communication). However, based on these data, it is likely that signals from the pharyngeal endoderm are both sufficient and required to induce the columella condensation.

### Chondrogenesis occurs in the columella earlier than in the extracolumella

Although the columella condensation is distinct by E6, the onset of chondrogenesis is not detected by Alcian blue staining in section or whole mount extractions until E7. Initially, chondrogenesis occurs in distinct chondrogenic centers beginning in the columella shaft and proceeds to two centers in the infracolumella arm before being detected in the supracolumella and extracolumella arms by E8. Chick Sox9 is expressed in pre-chondrogenic cells in nascent condensations at E5 and E6 and Sox9 expression is partially responsible for the differentiation of chondrocytes [21,23,28]. Bmp2 has a brief period of upregulation in the columella just as chondrogenesis occurs, with concomitant downregulation of Bmp4.

BMP is required for condensation proliferation and chondrogenesis [29], and our results show that Col2 expression is initiated at the onset of chondrogenesis. Overexpression of BMPs leads to increased cartilage (larger condensations, increased proliferation and differentiation), whereas loss of function has the opposite effect [29], indicating that BMPs, together with Sox9, are partially responsible for differentiation. However, in some cases such as the chick mandibular process, increased amounts of BMPs resulted in increased cell death and abnormal skeletogenesis [30]. Noggin upregulation also acts to restrict the effect of BMP, allowing for correct differentiation [14]. The context specific mechanism in columella chondrogenesis remains to be determined.

Although Sox9 is known to regulate Col2 expression, Col2 upregulation occurs much later than Sox9, at E7, which suggests a repressive mechanism is in place that modulates the effect of Sox9 expression and delays the onset of chondrogenesis and Col2 expression. With downregulation of Sox9, at E9, Col2 also downregulates, and Ihh expression is initiated, indicating that differentiation of chondrocytes toward hypertrophy has begun. Neither Col2 nor Ihh is expressed in the cells of the perichondrium between E7 and E10.

### Bone formation occurs in the replacement cartilage of the shaft and footplate

Osteogenesis of periosteum cells in the columella shaft occurs by E13.5, as determined by Alizarin Red S staining and COL1 immunohistochemistry. The identification of two separate domains of osteoblast cells--with positive cells distinguishable in the footplate a half-day later than in the shaft--was unexpected. These domains merge by E15 with osteoblasts invading the cartilage matrix via the periosteum layer and the periosteum of the shaft extending toward the extracolumella. The molecular mechanism that leads to final positioning of the jointing region between replacement and persistent cartilage remains unknown. However, co-expression of Sox9 and Runx2 are required for ossification of replacement cartilage, whereas tissue that expresses Sox9 alone will remain as persistent cartilage [31].

## Conclusion

We have determined the timing of morphogenesis and gene expression patterns of the middle ear forming region. These data are crucial for future studies elucidating the molecular mechanisms required to specify second arch regional identity, including columella condensation induction and regionalizing signals from the adjacent pharyngeal endoderm.

## Methods

Fertilized chicken eggs (Morgan Poultry Center, Clemson University) were incubated at 38.5°C in a humidified chamber until the desired stage was reached.

### Paraffin sectioning

Following fixation embryos were embedded by passing them through a graded series of alcohol, 2 × 30 mins Neo-Clear, 1:1 Neo-Clear/Paraplast, 3 × 30 min Paraplast under vacuum. Sectioning was performed at 10-12 μm. Slides were baked at 50°C for 1 hr, dewaxed in 2 × 5 min NeoClear, followed by graded ethanol to running water. Slides were then processed for in situ hybridization or immunostaining.

### In situ hybridization

Analysis of whole mount embryos was performed as previously described [32]. Section analysis (2-5 serially sectioned embryos, per stage, per gene) was performed by post fixing dewaxed paraffin sections for 10 mins in 4% PFA (paraformaldehyde)/PBS (Phosphate buffered saline), quenched for 20 mins 3% H_2_O_2_, 10 mins 1 × PBS, 5 mins active DEPC/H_2_O, then digested in 2.0 μg/ml Proteinase K for 5-15 mins depending on stage. Several PBS washes were followed by 30 mins permeabilization with PBS/1% Triton-X. Following several PBS rinses slides were dehydrated through a graded series of ethanol washes, with pre-hybridization buffer added at room temperature for 1-2 hours (0.25 ml 2 M DTT [dithiothreitol], 2 ml 10 mg/ml yeast RNA, 1 ml 10 mg/ml hsDNA, 1 ml 50 × Denhardt's solution, 10 ml 50% Dextran sulphate, 25 ml Formamide, 5 ml Salt solution, H_2_O to 50 ml). Hybridization buffer with DIG-labeled RNA probe was added at 60-70°C in a slide moat overnight. SSC (NaCl, Na citrate) washes (1 × and 0.5 ×) were performed twice for 30 mins at 60°C, followed by MABT (Maleic acid buffer/Tween-20) at room temperature 2 hr, 2% blocking reagent/MABT 1 hr, then anti-DIG-AP antibody (1:200) overnight at 4°C. Tissue was washed in MABT at room temperature for 1-2 hr, 10 min NTMT (NaCl, Tris, MgCl, Tween-20), and then staining was developed with NBT/BCIP (Nitro Blue Tetrazolium/5-Bromo-4-Chloro-3- Indolyl Phosphate). Following color development slides were rapidly dehydrated in EtOH, air-dried and coverslipped with Vectamount (Vector labs) and dried for 48 hr before imaging.

Accession number of probes used, with base pairs indicated. Bmp2: [NM_204358], 131-914; Bmp4: [NM_205237], 126-1075; Col2: [CHKC2A106], 200-1058; Ihh: [NM_204957], 2-557; Sox9: [U12533], 199-1544.

### Immunohistochemistry

Labeling was performed by incubating dewaxed slides in 4 μg/ml Proteinase K for 15 min at 37°C, 2 × 2 mins PBS, 3 × 5 mins PBT (PBS/0.1% triton-X100). Blocking with PBT (PBT plus 5% goat serum/0.2% BSA [bovine serum albumin]) for 1 hr, followed by primary antibody overnight at 4°C (1:300 COL1, mouse monoclonal, Abcam-ab6308). Slides were washed 3 × 5 min PBT, 1 hr secondary antibody (1:200, goat anti-mouse AlexaFluor 488), PBT 2 × 5 mins, PBS 5 mins. For fluorescence imaging, slides were mounted in Slowfade (Invitrogen).

Peanut agglutinin lectin (PNA) staining was performed with an initial peroxide step, 2 × 5 mins PBS, then 100 μl PNA solution (Sigma-L7759) at 4°C for 48 hr. PBT (PBS/0.1% Tween-20) 3 × 5 mins followed by DAB staining and visualization after mounting.

### Histological staining

For tissue sections slides were treated according to a protocol from P. Francis-West. Slides were dewaxed and rehydrated to water, dipped for 2 min in acetic alcohol (45 ml of 95% ethanol, 45 ml H_2_O, 10 ml glacial acetic acid), then stained in Alcian blue 10-20 min until color developed (stock: 1 g in 80% ethanol; dissolve working stain in acetic alcohol to desired strength). Slides were rinsed in acetic alcohol for 2 min, washed in running water, treated with 1% phosphomolybdic acid for 5-10 min, again rinsed in running water and finally stained in 0.5% Chlorantine fast red (Direct Red 81 can substitute) for 10-15 min. Slides were rapidly dehydrated and mounted using Vectamount.

For columella extractions, whole heads were fixed in 95% EtOH for 7 days, followed by 15 mg/100 ml Alcian blue in acid alcohol (1:4 glacial acetic acid/95% EtOH) for 1-3 days depending on stage. Tissue was then washed 1-2 hr in 95% EtOH, then 0.5% KOH over night, clearing in 1% KOH for a further 1-2 days. If required, Alizarin Red S 7.5 mg/100 ml H_2_O for 1-3 days for bone staining. Then cleared again with KOH in a graded series with glycerol until clearing is sufficient to identify and extract the columella. Long-term storage in 1:1 glycerol/H_2_O at 4°C.

## Authors' contributions

JLW performed and analyzed the ISH markers. AJH performed and analyzed all the immunolabeling. KJM performed and analyzed the ISH and staining. SCC performed the staining analysis, conceived and designed the study and interpreted the data. All authors were responsible for drafting the manuscript. All read and approved the final manuscript.
